# Synthesis, molecular docking, and in vivo antidiabetic evaluation of new benzylidene-2,4-thiazolidinediones as partial PPAR-γ agonists

**DOI:** 10.1038/s41598-023-47157-x

**Published:** 2023-11-14

**Authors:** Asim Najmi, Md Shamsher Alam, Neelaveni Thangavel, Manal M. E. Taha, Abdulkarim M. Meraya, Mohammed Albratty, Hassan A. Alhazmi, Waquar Ahsan, Anzarul Haque, Faizul Azam

**Affiliations:** 1 Department of Pharmaceutical Chemistry, College of Pharmacy, Jazan University, P. Box No. 114, Jazan, Saudi Arabia; 2 Substance Abuse and Toxicology Research Centre, Jazan University, P. Box No. 114, Jazan, Saudi Arabia; 3 Pharmacy Practice Research Unit, Department of Clinical Pharmacy, College of Pharmacy, Jazan University, Jazan, Saudi Arabia; 4 Medical Research Center, Jazan University, Jazan, Saudi Arabia; 5 Department of Pharmaceutics, Buraydah College of Dentistry and Pharmacy, P.O Box 31717, Buraydah, Al-Qassim Saudi Arabia; 6 Department of Pharmaceutical Chemistry and Pharmacognosy, Unaizah College of Pharmacy, Qassim University, Unaizah, Saudi Arabia

**Keywords:** Drug discovery, Drug screening, Medicinal chemistry

## Abstract

Peroxisome proliferator-activated receptor-γ (PPAR-γ) partial agonists or antagonists, also termed as selective PPAR-γ modulators, are more beneficial than full agonists because they can avoid the adverse effects associated with PPAR-γ full agonists, such as weight gain and congestive heart disorders, while retaining the antidiabetic efficiency. In this study, we designed and synthesized new benzylidene-thiazolidine-2,4-diones while keeping the acidic thiazolidinedione (TZD) ring at the center, which is in contrast with the typical pharmacophore of PPAR-γ agonists. Five compounds (**5a–e**) were designed and synthesized in moderate to good yields and were characterized using spectral techniques. The in vivo antidiabetic efficacy of the synthesized compounds was assessed on streptozotocin-induced diabetic mice using standard protocols, and their effect on weight gain was also studied. Molecular docking and molecular dynamics (MD) simulation studies were performed to investigate the binding interactions of the title compounds with the PPAR-γ receptor and to establish their binding mechanism. Antidiabetic activity results revealed that compounds **5d** and **5e** possess promising antidiabetic activity comparable with the standard drug rosiglitazone. No compound showed considerable effect on the body weight of animals after 21 days of administration, and the findings showed statistical difference (*p* < 0.05 to *p* < 0.0001) among the diabetic control and standard drug rosiglitazone groups. In molecular docking study, compounds **5c** and **5d** exhibited higher binding energies (− 10.1 and − 10.0 kcal/mol, respectively) than the native ligand, non-thiazolidinedione PPAR-γ partial agonist (nTZDpa) (− 9.8 kcal/mol). MD simulation further authenticated the stability of compound **5c**-PPAR-γ complex over the 150 ns duration. The RMSD, RMSF, rGyr, SASA, and binding interactions of compound **5c**-PPAR-γ complex were comparable to those of native ligand nTZDpa-PPAR-γ complex, suggesting that the title compounds have the potential to be developed as partial PPAR-γ agonists.

## Introduction

One of the major growing threats for the human population worldwide is diabetes mellitus (DM), and a considerable percentage of human population is currently suffering from type 1 or 2 DM^1^. Although several antidiabetic drugs of different classes are available in the market, most of them are associated with minor to serious side effects^2,3^. One of the drug classes used for type 2 DM is thiazolidinediones (TZDs) or glitazones, which act as insulin sensitizers. They regulate the transcription of genes responsible for glucose and lipid metabolism via binding to the peroxisome proliferator-activated receptor-γ (PPAR-γ)^4^. Through PPAR-γ stimulation, glitazones also regulate cell development, angiogenesis, and inflammation, which broadens their restorative potential^4^.

The positive correlation between adipogenesis and PPAR-γ action signifies the undesirable weight gain linked to the use of TZDs in people with DM and obesity^5,6^. TZDs have also been reported to have other serious adverse effects, such as cardiotoxicity, congestive heart failure, and even conceivable carcinogenesis in a few cases^7,8^. Hence, the pleiotropic effects of TZDs must be clarified to ensure medication safety and facilitate novel drug design and development. Selective PPAR-γ modulators (SPPARMs), also known as the partial agonists/antagonists of PPAR-γ subtype, disengage insulin sensitization from triglyceride (TG) accumulation in people with type 2 DM^9^. SPPARMs also help avoid the unfavorable effects of using full agonists, such as body weight gain and cardiotoxicity.

Several new derivatives with or without the TZD ring system have been designed and tested for their partial agonistic activities on PPAR-γ, resulting in the identification of various pharmacophores that could bind partially at the binding site of the protein^10,11^. In this study, we designed new benzylidene-2,4-thiazolidinediones by keeping the acidic polar head in between the lipophilic ring and the linker instead of placing it at the end, which is typical for full agonists. This new strategy was expected to have effect on the binding to the receptor, which was studied using molecular docking by investigating the plausible interactions between the molecule and protein. The antidiabetic potential of the newly synthesized derivatives was assessed using an in vivo antidiabetic rodent model.

## Materials and methods

### Chemicals, reagents, and instruments

All the chemicals, reagents, and solvents used in this study were purchased from Sigma Aldrich (Steinheim, Germany) and used without further purification. The melting points of all synthesized compounds were measured by the open capillary method using a melting point apparatus (Stuart SMP30, Massachusetts, USA). The infrared (IR) spectra of the compounds were recorded using a Fourier transform-infrared spectrometer (FT-IR) coupled with attenuated total reflectance (FTIR–ATR) (Nicolet iS10, Thermo Scientific, Massachusetts, USA). The nuclear magnetic resonance (NMR) spectra of the synthesized compounds were obtained using NMR spectrometer (Bruker 500 Ultra shield, Massachusetts, USA) operating at 500 MHz frequency for ^1^H NMR and 100 MHz frequency for ^13^C NMR analysis using dimethyl sulfoxide (DMSO) as the solvent and tetramethylsilane as the internal standard. The mass spectra (MS) were recorded using a triple quadrupole mass spectrometer (ThermoFisher Scientific, Massachusetts, USA). The completion of reactions was monitored using thin-layer chromatography (TLC) on precoated (F-254) aluminum-supported TLC plates (Merck, Darmstadt, Germany). Benzene: methanol (9:1) was used as the solvent system, and the retardation factor (*R*_*f*_) was calculated.

### Animals

In vivo studies were performed on male Swiss albino mice obtained from the central animal house facility, Jazan University, Saudi Arabia and were kept in the animal house at the College of Pharmacy until experiments were performed. All the animals were acclimatized for 1 week under standard conditions with a temperature of 22 ± 1 °C and humidity of 50% ± 5% with a 12 h light/dark cycle and had free access to commercial food pellets and fresh tap water. The experimental protocol was approved by the Standing Committee for Scientific Research Ethics-Jazan University (HAPO-10-Z-001) prior to conducting the experiments with an approval number REC42/1/027. All the procedures were in accordance with the guidelines set by the Organization for Economic Cooperation and Development and International Conference on Harmonization. The results of the experiments were reported in accordance with the ARRIVE 2.0 guidelines.

### Synthesis of titled compounds

**Figure 1:**
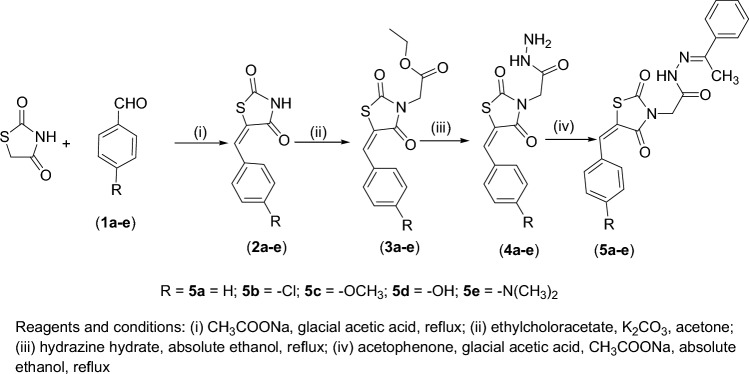
Synthetic route to the titled compounds (**5a–e**).

All the titled compounds were synthesized using the scheme shown in Fig. 1.

#### Synthesis of 5-(4-substituted benzylidene)-thiazolidine-2,4-diones (2a–e)

Thiazolidine-2,4-dione (0.25 mol) and an appropriate proportion of aryl aldehydes (0.25 mol) were mixed in 50 mL of hot glacial acid, followed by the addition of fused sodium acetate (1.8 g) to the resulting solution. The reaction mixture was then refluxed for 5 h. The progress of the reaction was continuously monitored using TLC. Upon the completion of the reaction, the reaction mixture was allowed to cool at room temperature and then added to ice-cold water. Thus, a solid product was obtained, filtered, dried, and purified by recrystallization from glacial acetic acid.

#### Synthesis of [5-(4-substituted-benzylidene)-2,4-dioxothiazolidin-3-yl]acetic acid ethyl esters (3a–e)

Substituted-benzylidene thiazolidine-2,4-diones (**2a–e**, 0.02 mol), ethyl chloroacetate (0.04 mol), and potassium carbonate (0.04 mol) were mixed in acetone (250 mL) and refluxed for 16 h. The inorganic base was filtered off, and the filtrate was evaporated to dryness under reduced pressure using a rotavapor. The obtained solid residue was recrystallized using methanol to afford the corresponding acetates (**3a–e**).

#### Synthesis of [5-(4-substituted-benzylidene)-2,4-dioxothiazolidin-3-yl]acetic acid hydrazides (4a–e)

Substituted-benzylidene thiazolidine-2,4-dione ethyl esters (**3a–e**, 0.01 mol) were added to hydrazine hydrate (10 mL), then mixed with an appropriate quantity of absolute ethanol (30 mL), and stirred to achieve a clear solution. The resulting solution was refluxed for 7 h. After the reaction was completed, ethanol was concentrated under vacuum to obtain the crude compound, which was then stirred into *n*-hexane, filtered at reduced pressure, and dried. Finally, the obtained desired compound was purified by recrystallization under ethanol.

#### Synthesis of [5-(4-substituted-benzylidene)-2,4-dioxothiazolidin-3-yl]acetic acid (1-phenylethylidene)hydrazides (5a–e)

Equimolar quantities (6 mmol) of substituted-benzylidene thiazolidine -2,4-dione hydrazides (**4a–e**) and acetophenone were dissolved in absolute ethanol (50 mL), followed by the addition of glacial acetic acid (3 mL). The resulting mixture was then refluxed for 10 h, and the progress of the reaction was monitored by TLC using ethyl acetate and petroleum ether (2:1) as solvents. After the reaction was completed, the reaction mixture was cooled to room temperature, concentrated under reduced pressure, and recrystallized using ethanol to afford the corresponding hydrazides as titled compounds (**5a–e**).

#### (5-Benzylidene-2,4-dioxo-thiazolidin-3-yl)-acetic acid (1-phenyl-ethylidene)-hydrazide (5a)

Color: light brown; Yield: 38%; Melting point (MP): 120–122 °C. FT-IR (KBr, ν, cm^−1^): 3244(–NH), 3058(Ar–CH), 3005 (CH), 2925(CH_2_), 1705, 1659, 1602 (C=O), 1556 (C=C), 1492 (C=N); ^1^H NMR (500 MHz, DMSO-*d*_6_, δ ppm): 1.72 (s, 3H, CH_3_), 4.13 (s, 2H, CH_2_), 7.10 (s, 1H, CONH), 7.46 (s, 1H, methylidene C–H), 7.96–7.05 (m, 10H, Ar–H); ^13^C NMR (100 MHz, DMSO-*d*_6_, δ ppm): 15.9 (CH_3_), 40.36 (CH_2_), 126.94 (C=C, Thiazolidinedione), 127.1, 127.2, 128.5, 128.7, 128.9, 129.0, 130.2, 130.9, 131.0, 131.3, 135.9, 137.2 (C, Benzene), 137.3 (methylidene C=C), 153.7, 156.2, 159.1 (C=O), 160.1 (C=N); Mass *m/z* (M + H)^+^: 380.15; Analysis calculated for C_20_H_17_N_3_O_3_S: C (63.31); H (4.52); N (11.07); O (12.65); S (8.45); *R*_*f*_ value = 0.75; Log P = 4.31.

#### [5-(4-Chloro benzylidene)-2,4-dioxo-thiazolidin-3-yl] acetic acid (1-phenyl-ethylidene)-hydrazide (5b)

Color: light brown; Yield: 35%; MP: 115–117 °C; FT-IR (KBr, ν, cm^−1^): 3334(–NH), 3193(Ar–CH), 3063(CH), 2924(CH_2_), 1774, 1682, 1604 (C=O), 1487 (C=C), 1443 (C=N), 759 (C–Cl); ^1^HNMR (500 MHz, DMSO-*d*_6_, δ ppm): 2.08 (s, 3H, CH_3_), 3.86 (s, 2H, CH_2_), 7.91–7.14 (m, 9H, Ar–H), 7.20 (s, 1H, CONH), 7.86 (s, 1H, methylidene C–H); ^13^CNMR (100 MHz, DMSO-*d*_6_, δ ppm): 15.6 (CH_3_), 40.19 (CH_2_), 126.93 (C=C, Thiazolidinedione), 127.1, 127.2, 128.7, 128.8, 120.0, 129.1, 130.2, 130.9, 131.0, 131.1, 131.3, 135.6 (C, Benzene), 135.7 (methylidene C=C), 153.7, 157.7, 159.1 (C=O), 160.06 (C=N); Mass *m/z* (M + H)^+^: 414.17; Analysis calculated for C_20_H_16_ClN_3_O_3_S: C (58.04); H (3.90); Cl (8.57); N (10.15); O (11.60); S (7.75); R_f_ value = 0.70; Log P = 5.02.

#### [5-(4-Methoxy benzylidene) -2,4-dioxo-thiazolidin-3-yl]-acetic acid (1-phenyl-ethylidene)-hydrazide (5c)

Color: deep brown; Yield: 40%; MP: 112–114 °C; FT-IR (KBr, ν, cm^−1^): 3343(–NH), 3063(Ar–CH), 2957(CH), 2921(CH_2_), 1723, 1683, 1604 (C=O), 1568 (C=C), 1490 (C=N), 1025(C–O–C); ^1^HNMR (500 MHz, DMSO-*d*_6_, δ ppm): 2.2 (s, 3H, CH_3_), 3.67 (s, 3H, OCH_3_), 3.88 (s, 2H, CH_2_), 7.92–7.04 (m, 9H, Ar–H), 7.11 (s, 1H, CONH), 7.84 (s, 1H, methylidene C–H); ^13^CNMR (100 MHz, DMSO-*d*_6_, δ ppm): 15.1 (CH_3_), 40.60 (CH_2_), 111.46 (C=C, Thiazolidinedione), 112.0, 112.2, 126.3, 126.92, 126.99, 128.6, 128.7, 128.8, 129.1, 129.9, 130.2, 133.6, 137.2 (C, Benzene), 138.3 (methylidene C=C), 152.5 (C=O), 157.7 (C=N); Mass *m/z* (M + H)^+^: 410.26; Analysis calculated for C_21_H_19_N_3_O_4_S: C (61.60); H (4.68); N (10.26); O (15.63); S (7.83); *R*_*f*_ value = 0.64; Log P = 4.23.

#### [5-(4-Hydroxy benzylidene) -2,4-dioxo-thiazolidin-3-yl]-acetic acid (1-phenyl-ethylidene)-hydrazide (5d)

Color: cream; Yield: 45%; MP: 110–112 °C; FT-IR (KBr, ν, cm^−1^): 3190(–OH), 3100(–NH), 2950(Ar–CH), 2924(CH), 2849(CH_2_), 1720, 1650, 1606 (C=O), 1507 (C=C), 1434 (C=N); ^1^HNMR (500 MHz, DMSO-*d*_6_, δ ppm): 1.6 (s, 3H, CH_3_), 2.57 (s, 2H, CH_2_), 4.12 (s, 1H, OH), 7.90–7.07 (m, 9H, Ar–H), 7.4 (s, 1H, CONH), 7.73 (s, 1H, methylidene C–H); ^13^CNMR (100 MHz, DMSO-*d*_6_, δ ppm): 21.9 (CH_3_), 40.1 (CH_2_), 116.17 (C=C, Thiazolidinedione), 115.9, 116.17, 116.5, 122.3, 123.5, 127.12, 131.6, 131.7, 132.0, 132.6, 134.2, 139.0 (C, Benzene), 147.9 (methylidene C=C), 160.3, 165.2, 167.3 (C=O), 174.29 (C=N); Mass *m/z* (M + H)^+^: 396.19; Analysis calculated for C_20_H_17_N_3_O_4_S: C (60.75); H (4.33); N (10.63); O (16.18); S (8.11); *R*_*f*_ value = 0.60; Log P = 3.64.

#### [5-(4-Dimethylamino benzylidene) -2,4-dioxo-thiazolidin-3-yl]-acetic acid (1-phenyl-ethylidene)-hydrazide (5e)

Color: deep brown; Yield: 50%; MP: 118–120 °C; FT-IR (KBr, ν, cm^−1^): 3190(–NH), 2975(Ar–CH), 2914(CH), 2801(CH_2_), 1760, 1679, 1601 (C=O), 1520 (C=C), 1442 (C=N), 1227 (C–N); ^1^HNMR (500 MHz, DMSO-*d*_6_, δ ppm): 2.26 (s, 3H, CH_3_), 2.91 (s, 6H, N-CH_3_), 4.12 (s, 2H, CH_2_), 7.96–7.01 (m, 9H, Ar–H), 7.4 (s, 1H, CONH), 7.70 (s, 1H, methylidene C–H); ^13^CNMR (100 MHz, DMSO-*d*_6_, δ ppm): 21.9 (CH_3_), 40.1 (CH_2_), 39.8, 39.9 (N-CH_3_), 111.6 (C=C, Thiazolidinedione), 112.9, 113.7, 115.3, 115.9, 116.1, 123.5, 126.8, 127.1, 127.3, 128.5, 128.7, 128.8 (C, Benzene), 131.7 (methylidene C=C), 144.5, 153.9, 155.0 (C=O), 167.0 (C=N); Mass *m/z* (M + H)^+^: 423.23; Analysis calculated for C_22_H_22_N_4_O_3_S: C (62.54); H (5.25); N (13.26); O (11.36); S (7.59); *R*_*f*_ value = 0.60; Log P = 4.47.

### Acute oral toxicity test

The acute oral toxicity test of the titled compounds was performed in vivo on male Swiss albino mice (24 ± 2.5 g) using a reported procedure^12,13^. The mice were fasted for 4 h prior to the administration of the test compounds. The solubility profile of the test chemicals at various dosage levels was determined, and a vehicle consisting of 5% DMSO and distilled water was used. The test chemicals were administered orally at consecutive doses beginning at 10 mg/kg. The second dosage of 50 mg/kg was administered after 48 h, and the animals were observed for symptoms of toxicity, particularly in the first 4 h. The dosage was further increased to 500 mg/kg, and the survival of animals was monitored for 14 days. The test mice were observed for acute poisoning symptoms such as lacrimation, hair erection, blinking, increased urine output, muscular weakness, drowsiness, convulsions, decrease in motor activity, diarrhea, coma, and death.

### Oral glucose tolerance test

Oral glucose tolerance test (OGT) was performed on male Swiss albino mice weighing 24 ± 2.5 g^14^. After a 16 h fast, each mouse had their blood glucose level (BGL) checked, and those with BGLs between 60 and 90 mg/dL were divided into 12 groups of six mice each. The mice in the experimental groups were given oral doses of 50 mg/kg and 100 mg/kg body weight of synthesized chemicals dissolved in 5% DMSO. The same amount of DMSO was given to the test, positive control, and negative control group. Rosiglitazone (12.5 mg/kg body weight) was administered orally to the positive control group. A glucose load (2.5 g/kg body weight) was given orally to each animal 30 min after the test sample or vehicle was administered. The blood glucose profile of each mouse was measured using a glucometer at 0, 30, 60, 90, and 120 min after glucose administration. The BGL of each mouse was assessed three to four times, and the average was calculated. During the trial, the cages were devoid of food but not of water. OGT was used to screen the test compounds for hypoglycemic action in normal, healthy mice at two dosage levels (50 and 100 mg/kg body weight) to establish the therapeutic dose. For future studies, the dosage that improves glucose tolerance in a similar way to the standard drug was chosen.

### In vivo antidiabetic activity

DM was induced in the mice through a single intraperitoneal (i.p.) injection of nicotinamide and streptozotocin (STZ)^15,16^. First, nicotinamide was dissolved in normal saline and administered at a dose of 120 mg/kg body weight into the mice fasted overnight. At 15 min post nicotinamide administration, STZ was injected intraperitonially at 60 mg/kg body weight dose. The STZ solution (0.1 M, pH 4.5) was prepared fresh by dissolving an appropriate amount of STZ in citrate buffer. The fasting blood glucose (FBG) level was measured after 72 h in each mouse by taking the blood samples from the tail vein using a glucometer. Each mouse’s BGL was monitored three to four times, and only those with average BGL over 200 mg/dL were chosen for the study and designated as STZ-induced diabetic animals.

The selected STZ-induced diabetic mice were weighed again before the study and split randomly into seven groups with six animals each. Group 1, the diabetic control group, was orally given 5% DMSO only as vehicle. Group 2 served as the positive control group and was given the standard drug rosiglitazone. Groups 3–7 orally received the synthesized compounds (**5a–e**) dissolved in DMSO. All the animals were fasted overnight, and their FBG levels on day 0 were determined. The test compounds were administered individually to the assigned groups at a dose of 100 mg/kg orally every day at a fixed time for 21 days. The vehicle was given to the negative control group, and rosiglitazone was administered at a dosage of 12.5 mg/kg body weight to the positive control group. Blood samples were withdrawn on days 0, 7, 14, and 21 from all the animals, and the percentage decrease in FBG was determined. On each sampling day, blood samples were collected from the tail vein of each mouse 3–4 times, and the average FBG was calculated.

### Effect on body weight

For 21 days, all the test compounds were orally administered at 100 mg/kg dose, and standard rosiglitazone was administered at 12.5 mg/kg daily at the same time to the diabetic mice. Their effects on body weight was studied relative to the body weight of the diabetic control.

### Molecular docking study

The 3D structure of PPAR-γ bound with its partial agonist nTZDpa (PDB ID: 2Q5S) was downloaded from the Research Collaboratory for Structural Bioinformatics database (https://www.rcsb.org). Chain A was deleted, and chain B was used for processing the receptor structure for docking. The binding coordinates of the native ligand nTZDpa were marked and retained for binding site identification. After the removal of native ligand and solvent, the receptor was energy-minimized by applying Amber ff14SB force field for standard residues and Gasteiger charges for other residues in Chimera 1.13.1^17^. The prepared receptor was saved in *.pdbqt format for further use. The structures of ligands under investigation were drawn in ChemDraw and saved in protein data bank (PDB) format. The native ligand nTZDpa structure for redocking was retrieved from PubChem in SMILES format. All ligand structures were processed in Chimera to obtain a minimum energy conformer through 100 steepest descent steps with the step size of 0.02 Å, followed by 10 conjugate gradient steps of size 0.02 Å. The stabilized ligand conformers were also saved in *.pdbqt format.

Molecular docking was carried out in Chimera-based AutoDock Vina. The docking grid was set up around the binding site. This grid box has dimensions centered at 35 × 8 × 40 Å and a size 20 × 20 × 20 Å corresponding to the X, Y, and Z axes. The docking protocol searched for a maximum of 10 conformers per ligand at an energy difference of 3 kcal/mol, with exhaustiveness of search set at 8. Ligand conformers were then ranked according to their predicted binding energy. The conformer with the minimum binding energy was chosen as the best conformer and retained for further binding interaction analysis. The investigated ligands were manually ordered based on their binding energies: the compound with the highest negative value for binding was the best active compound. The reliability of the developed docking protocol was verified by redocking the native ligand nTZDpa to 2Q5S. The standard deviation between the conformers of the native ligand and the redocked native ligand was an indication of reliability: the lower the root-mean-square deviation (RMSD), the higher the reliability. The docked complexes of 2Q5S were probed for the intermolecular binding interactions in Discovery Studio.

### Protein–ligand interaction-based pharmacophore model analysis

The 3D pharmacophore model was analyzed with LigandScout 4.3 software using the 3D structures of 2Q5S docked to compounds **5c** and **5d**. The 3D pharmacophore model was generated for compound **5c** first by loading the corresponding docked complex onto the macromolecular view. The ligand was then checked for missing bonds, if any, by focusing on the yellow box on the ligand. Afterward, the 3D pharmacophore model was created by selecting the create model option. The ligand and the pharmacophore model were saved to the alignment mode. The 3D structure of 2Q5S complexed with compound **5d** was then loaded, and the procedure was repeated. Both pharmacophore models from compounds **5c** and **5d** were aligned, and the fingerprint features were shared and merged to create a 3D pharmacophore model with merged structural features^18^.

### Molecular dynamics simulation studies

MD simulation was used to examine the thermodynamic behavior and stability of the best-ranked conformation of compound 5c in contact with PPAR-γ provided by the docking study. We used the Desmond 7.4 software integrated with Maestro of Schrödinger, Inc. (https://www.schrodinger.com/products/desmond) for MD. To better understand the conformational changes caused by the test ligand binding, the apo form of the PPAR-γ and native partial agonist nTZDpa-PPAR-γ complex were also simulated. Inside an orthorhombic box with a 10 Å buffer region between protein atoms and box sides, the apo form of PPAR-γ and compound 5c-PPAR-γ or nTZDpa-PPAR-γ complexes were inserted. The box was then filled with the required number of water molecules in accordance with the system setup procedure.

A simple point charge (SPC) model and OPLS3e force field were adopted for the MD computations^19^. The system was neutralized with an appropriate number of Na^+^ and Cl^−^ ions, at a salt concentration of 0.15 M, which reflects monovalent ion concentration in the body. The temperature and pressure were set to 300 K and 1.01325 bar, respectively, using an isothermal-isobaric (NPT) ensemble. The simulation time was customized to 150 ns, and trajectories were recorded every 150 ps. For short-range van der Waals and Coulomb interactions, a cut-off radius of 9.0 was chosen. The system temperature and pressure were maintained using the Nose–Hoover thermostat and Martyna–Tobias–Klein techniques, respectively. For bonded and non-bonded interactions inside the short-range cut-off, the reversible reference system propagator algorithm (RESPA) integrator was employed with an inner time step of 2.0 fs to integrate the equations of motion. The system was minimized and equilibrated to Desmond's default protocols. The Desmond package's simulation interaction diagram module was used to examine the trajectory files.

### Statistical analysis

The data obtained for the antidiabetic activity using the in vivo STZ-induced diabetic model and the effect of synthesized compounds on the body weight were analyzed by two-way mixed analysis of variance (ANOVA) using RStudio program version 1.4.1564 (Posit, PBC, Vienna, Austria). The mean ± standard error of the mean (SEM) values were compared and cross-classified by two independent categorical variables, including one between the groups and one within the group. Values (*p*) < 0.05 were significant.

## Results and discussion

### Synthesis

New [5-(4-substituted-benzylidene)-2,4-dioxothiazolidin-3-yl]acetic acid (1-phenylethylidene)hydrazides (**5a–e)** were synthesized first by reacting thiazolidine-2,4-dione and appropriate aromatic aldehydes (**1a–e**) to afford compounds 5-(4-substituted-benzylidene) -thiazolidine-2,4-diones (**2a–e**). The corresponding ethyl esters (**3a–e**) were then synthesized by reacting benzylidene-thiazolidine-2,4-diones (**2a–e**) with ethyl chloroacetate. The substituted [5-(4-substituted-benzylidene)-2,4-dioxothiazolidin-3-yl] acetic acid hydrazides (**4a–e**) were synthesized by refluxing the ethyl esters (**3a–e**) with hydrazine hydrate. Finally, the target benzylidene-2,4-thiazolidinediones **(5a–e)** were synthesized by reacting the acetic acid hydrazides (**4a–e**) with acetophenone.

All the synthesized compounds were characterized using various spectral techniques such as FT-IR, ^1^H-NMR, ^13^C-NMR, and MS. The structures of all the synthesized compounds were supported by their physiochemical properties and spectral characteristics. The FT-IR spectra of the titled compounds showed characteristic bands at 3190 (− OH), 3343–3100 (− NH), 3193–2950 (Ar–CH), 3063–2914 (CH), 2925–2801 (CH_2_), 1774–1601 (C = O), 1568–1487 (C = C), 1492–1434 (C = N), 1227 (C-N), 1025 (C–O–C), and 759 (C–Cl). The ^1^H-NMR spectra confirmed the presence of − CH_3_, − CH_2_, CONH, and − C-H groups by showing singlets at δ 1.6–2.26, δ 2.57–4.13, δ 7.1–7.4, and δ 7.46–7.86 ppm, respectively. Meanwhile, the Ar–H groups were confirmed by detecting multiplets at δ 7.96–7.01 ppm. Singlets for − OCH_3_ and OH were observed at 3.67 and 4.12 ppm, respectively. The ^13^C-NMR spectra confirmed the presence of − CH_3_, − CH_2_, C = C (thiazolidinedione), C (benzene), methylidene (C = C), and (C = N). Electrospray ionization (ESI) in MS revealed the protonated molecular ions in the positive ion mode. Mass *m/z* (M + H)^+^ peaks at 380.15, 414.17, 410.26, 396.19, and 423.23 were observed for compounds **5a**, **5b**, **5c**, **5d**, and **5e**, respectively.

### Acute toxicity test

The acute oral toxicity test was performed to evaluate the acute physical, fatal, or behavioral changes caused by the newly synthesized compounds when administered orally. The synthesized benzylidene-2,4-thiazolidinedione (**5a–e**) derivatives were administered in a series of doses ranging 10–500 mg/kg, followed by the measurement of weight before and after the treatment. The mice were monitored for 14 days and exhibited no symptoms of harm. Furthermore, no deaths occurred throughout the follow-up study. No significant variation in mouse weight was observed pre- and post-treatment, indicating the median lethal dose (LD_50_) of each compound to be more than 500 mg/kg.

### Oral glucose tolerance test

The blood glucose lowering action of each benzylidene-2,4-thiazolidinedione (**5a–e**) derivative was evaluated at 50 and 100 mg/kg doses in normal mice, and the results are summarized in Table 1. The blood glucose concentration was measured at six time points: 30 min before administering the test compounds, immediately before the glucose loading (0 min), and 30, 60, 90, and 120 min after the glucose administration. Compared with the control, all the tested compounds exhibited remarkable reductions in BGL at both doses (Table 1). All the tested compounds and the standard drug rosiglitazone caused a steady decrease in BGL as evident from the increasing percentage decrease in BGL with time. In particular, compound **5e** at 100 mg/kg dose showed the greatest percent decrease (59.79%) in mice at 120 min. This percent decrease was even higher than that for the standard drug rosiglitazone (41.90%) tested at 12.5 mg/kg dose after 120 min. Table 1 shows that the postprandial glucose-lowering effects of all the tested compounds were dose and time dependent.

**Table 1:** Postprandial glucose lowering effects (%) of test compounds on normal mice post-glucose load.

Group	Dose (mg/kg)	Percent decrease in BGL				
		0 min	30 min	60 min	90 min	120 min
Rosiglitazone	12.5	8.0 ± 1.4	35.2 ± 3.8	36.9 ± 3.9	39.4 ± 2.7	41.9 ± 3.9
**5a**	50	3.3 ± 0.5	28.4 ± 3.1	35.4 ± 4	38.7 ± 3.8	41.5 ± 2.2
	100	6.78 ± 0.9	29.8 ± 3.3	38.7 ± 3.9	43.1 ± 0.7	45.2 ± 1.5
**5b**	50	1.9 ± 0.5	18.5 ± 4.4	19.6 ± 4.6	20 ± 4.7	22.5 ± 4.1
	100	3.8 ± 0.3	19.6 ± 1.2	24.2 ± 1.8	25.4 ± 1.9	27.8 ± 1.5
**5c**	50	2.6 ± 0.2	22.1 ± 3.4	32.4 ± 3.7	38.4 ± 2.8	43.4 ± 2.9
	100	5.9 ± 0.7	28.4 ± 3.6	42.6 ± 3.7	47.2 ± 3.5	48.8 ± 3.9
**5d**	50	1.0 ± 0.2	13.9 ± 1.3	17.9 ± 2.3	19.4 ± 1.9	22.0 ± 2.5
	100	1.9 ± 0.9	18.7 ± 1.7	24.8 ± 2.5	26.4 ± 2.3	30.1 ± 2.8
**5e**	50	6.5 ± 1.0	30.7 ± 3.4	34.2 ± 4.2	40.3 ± 2.3	44.8 ± 3.6
	100	7.4 ± 1.2	43.9 ± 2.8	45.4 ± 4.2	53.3 ± 12.3	59.8 ± 16.6

### In vivo antidiabetic activity

The in vivo antidiabetic screening of synthesized benzylidene-2,4-thiazolidinediones (**5a–e**) were performed on the STZ-induced diabetic murine model. The test compounds (100 mg/kg) and rosiglitazone (12.5 mg/kg) were administered to the diabetic mice for 21 days at a set time each day. FBG levels were measured at the same time on days 0, 7, 14, and 21, and the findings are presented in Table 2. Compared with the control, all the test compounds caused a substantial decrease in FBG on days 7, 14, and 21 in a manner similar to the standard drug rosiglitazone. As evident from the lowest FBG, the maximum antidiabetic activity as was observed at day 21 for all the test compounds and rosiglitazone. Among the test compounds, the best antidiabetic activity was discovered for compound **5d** (% decrease = 59.28%), followed closely by **5e** (% decrease = 55.67%).

**Table 2:** Effect of test compounds (**5a–e**) and rosiglitazone on the FBG of STZ-induced diabetic mice.

Group	FBG (mg/dL)				% change (Day 21–Day 0)
	Day 0	Day 7	Day 14	Day 21	
Diabetic control	265.7 ± 17.2	302.9 ± 18.9	319.9 ± 24	345.7 ± 12.4	30.1 ± 13.4
Rosiglitazone	269.7 ± 2.1	191.0 ± 9.5****	161.7 ± 7.8****	130.7 ± 1.5****	51.5 ± 0.7
**5a**	287.0 ± 6.1*	195.7 ± 7.0****	167.0 ± 3.6****	132.3 ± 5.5****	53.9 ± 1.7
**5b**	273.3 ± 6.8	206.0 ± 14.7****	174.3 ± 10.2****	125 ± 6.6****	54.2 ± 3.0
**5c**	272.3 ± 16.9	210.0 ± 15.7****	164 ± 19.1****	124.3 ± 5.7****	54.4 ± 4.8
**5d**	298 ± 14**	194.3 ± 5.0****	158.7 ± 9.5****	121.3 ± 4.5****	59.3 ± 1.8
**5e**	285.0 ± 8.5	207.3 ± 16.8****	169.0 ± 2.7****	126.3 ± 5.5****	55.7 ± 1.0

*p < 0.05; **p < 0.01; ***p < 0.001; ****p < 0.0001.

**Figure 2:**
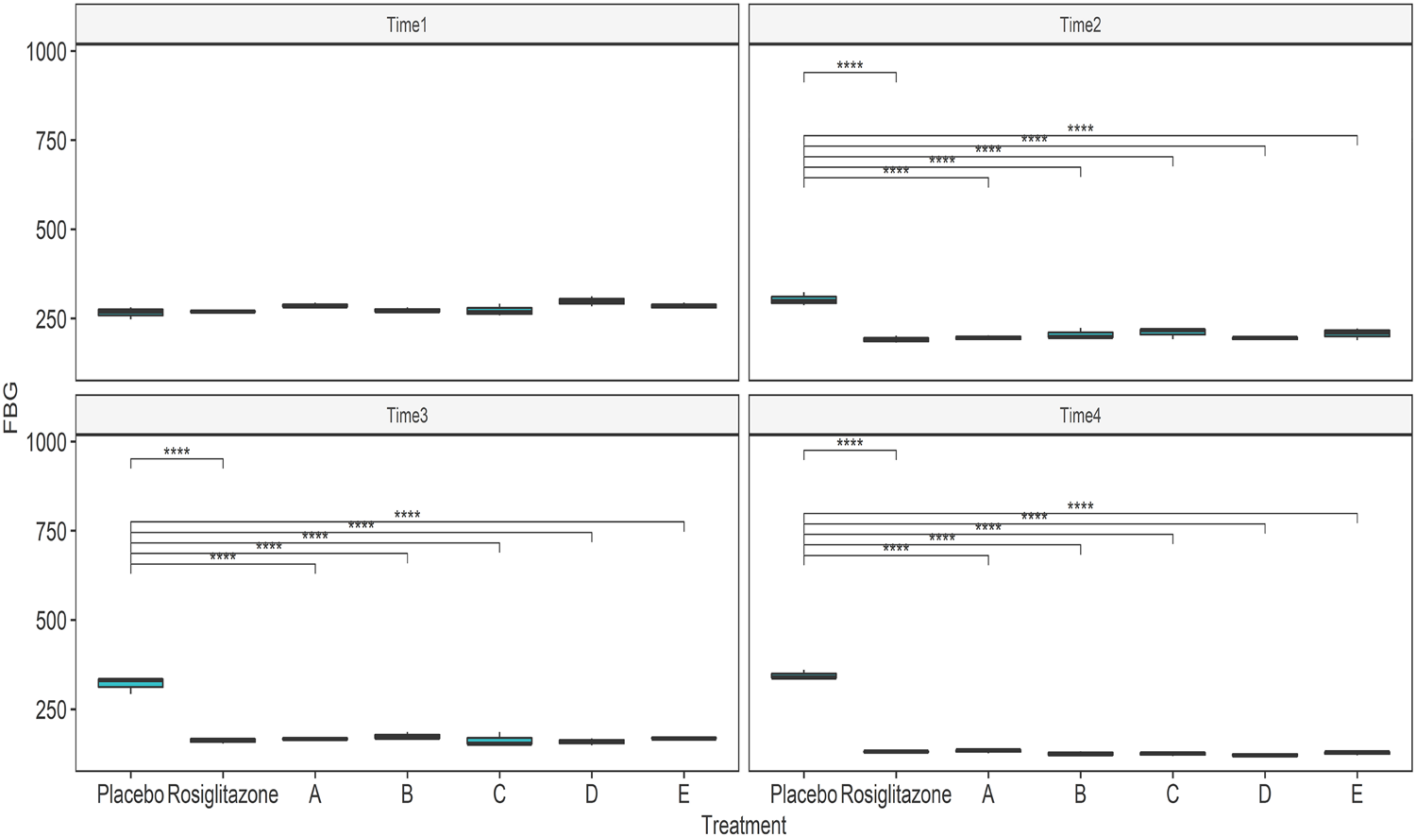
Pairwise comparison between different time points within treatment groups. Time 1, Time 2, Time 3, and Time 4 represents 0, 7, 14, and 21 days respectively; placebo = diabetic control; treatment A, B, C, D, and E represent test compounds **5a**, **5b**, **5c**, **5d**, and **5e**, respectively; ****p < 0.0001.

**Figure 3:**
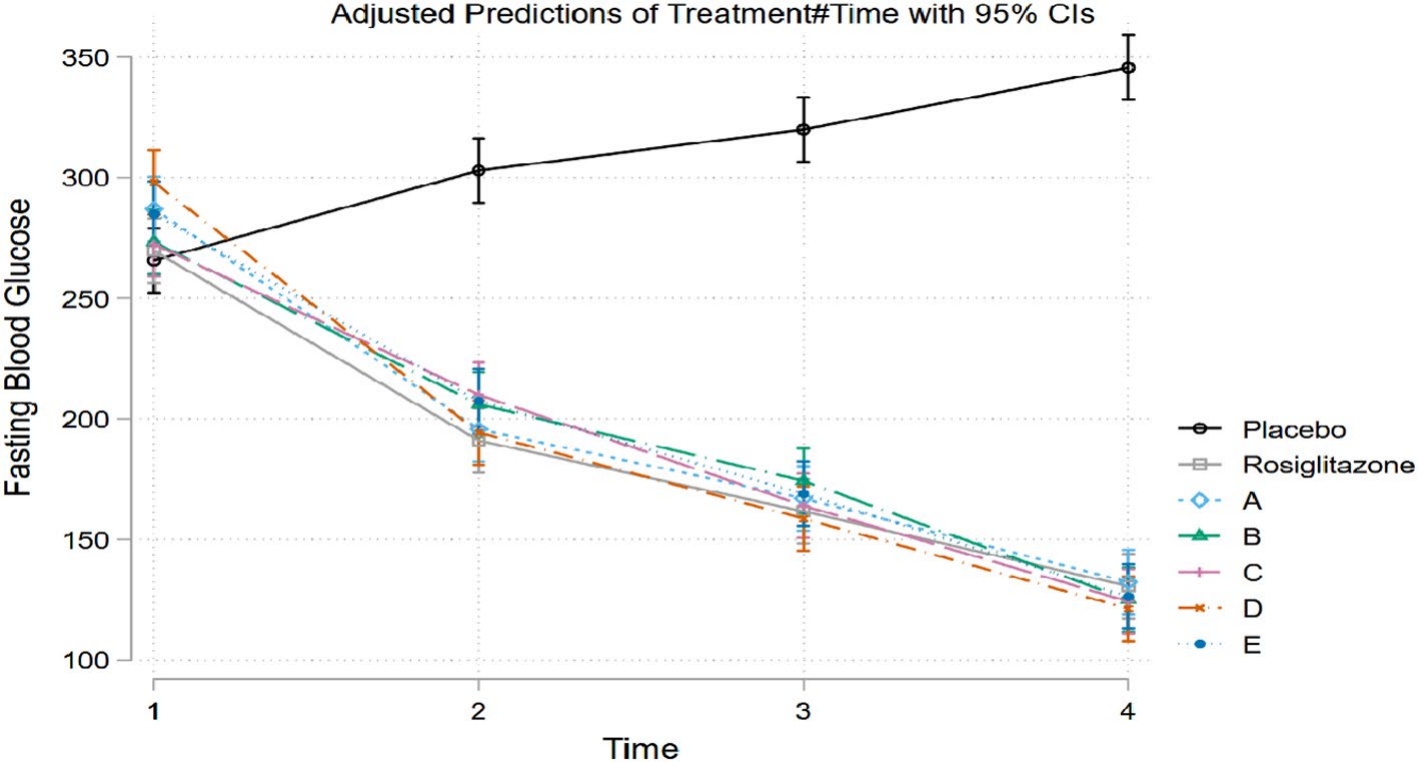
Graph showing effects of standard rosiglitazone and test compounds in FBG in comparison to the control. Placebo = diabetic control; treatment A, B, C, D, and E represent test compounds **5a**, **5b**, **5c**, **5d**, and **5e**, respectively.

Statistically significant two-way interactive effects were observed between treatment group and time on FBG with F(18, 42) = 31.4 and *p* < 0.0001. Pairwise comparison between groups at each time revealed that all the test compounds induced a highly significant (*p* < 0.0001) reduction in BGL comparable with the standard drug rosiglitazone. The maximum percent change in FBG level was observed with compounds **5d** (59.28%) and **5e** (55.67%). Furthermore, a pairwise comparison was performed between different times within groups to investigate the effects of treatment and time on the FBG, and the findings are depicted in Figs. 2 and 3. The mean FBG values were significantly different in the control versus treatment groups at all time points.

### Effects on the body weight of STZ-induced diabetic mice

The effects of the test compounds (**5a–e**) and the standard drug rosiglitazone on the body weight of STZ-induced diabetic animals were studied for 21 days, and the results are presented in Table 3. Rosiglitazone induced a slight increase in body weight at the end of the study period, and all the test compounds did not cause any changes in body weight. This finding showed that the synthesized compounds sdid not cause any considerable weight gain in the treated animals.

**Table 3:** Effects of test compounds on the body weight of STZ-induced diabetic mice.

Group	Body weight (g)				% change (Day 21–Day 0)
	Day 0	Day 7	Day 14	Day 21	
Diabetic control	25.0 ± 0.6	24.8 ± 0.3	23.7 ± 0.3	22.3 ± 0.9	–10.9 ± 1.2
Rosiglitazone	25.5 ± 0.4	26.0 ± 0.67*	26.8 ± 0.4****	27.5 ± 0.5****	7.7 ± 0.8
**5a**	25 ± 0.6	25.3 ± 0.4	26.0 ± 0.4**	25.9 ± 1.0***	3.5 ± 0.4
**5b**	24.6 ± 0.3	24.5 ± 0.2	24.0 ± 0.1	23.8 ± 0.2	−3.3 ± 0.5
**5c**	24.9 ± 0.3	24.6 ± 0.3	24.7 ± 0.4*	24.3 ± 0.4*	−2.5 ± 0.5
**5d**	24.2 ± 0.3	24.5 ± 0.2	24.7 ± 0.1*	25.1 ± 0.1**	3.5 ± 0.7
**5e**	24.7 ± 0.2	24.6 ± 0.2	24.8 ± 0.3*	24.8 ± 0.2**	0.9 ± 0.2

*p < 0.05; **p < 0.01; ***p < 0.001; ****p < 0.0001.

**Figure 4:**
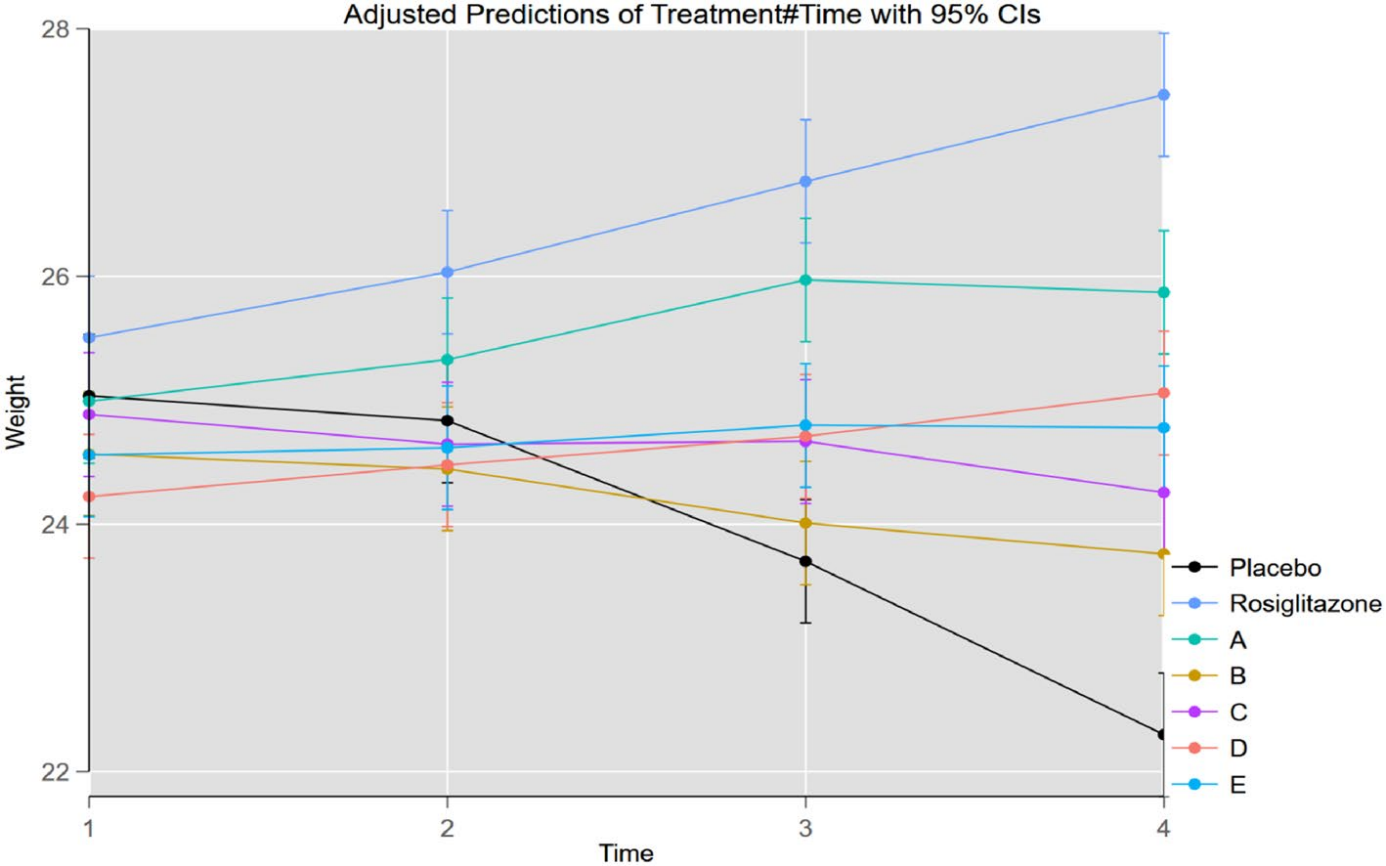
Change in animal body weight in different groups at time 1 (day 1), time 2 (day 7), time 3 (day 14) and time 4 (day 21). Placebo = diabetic control; treatment A, B, C, D, and E represent test compounds **5a**, **5b**, **5c**, **5d**, and **5e**, respectively.

The results of treated groups were statistically compared with those of the diabetic control group and the change in body weight was compared at each time point within and between the groups. The adjusted predictions are shown in Fig. 4**,** where the changes in the body weight in each group of animals are also presented. The untreated diabetic control mice showed a decreased body weight throughout the tested period, and those treated with the standard drug rosiglitazone showed an increased body weight.

The change in body weight induced by the test compounds was examined relative to the body weight of the control at different time points to determine the level of significance. None of the test compounds induced any significant difference in body weight compared with that of the control after 7 days; however, rosiglitazone caused a statistically significant (*p < 0.05) change in body weight relative to that of the control at this time. This effect continued after 14 days, with rosiglitazone inducing a highly significant (****p < 0.0001) change in body weight due to its opposite effects on body weight over time. For the test compounds, compound **5a** caused a significant (**p < 0.01) change in body weight relative to the control after 14 days. Compounds **5c**, **5d,** and **5e** also induced lesser but statistically significant changes in body weight (*p < 0.05) compared with that of the control. At the end of the study period (21 days), the level of significance for compounds **5d** and **5e** increased (**p < 0.01), and a similar trend was observed for compound **5a** (***p < 0.001) after 21 days.

### Molecular docking study

**Figure 5:**
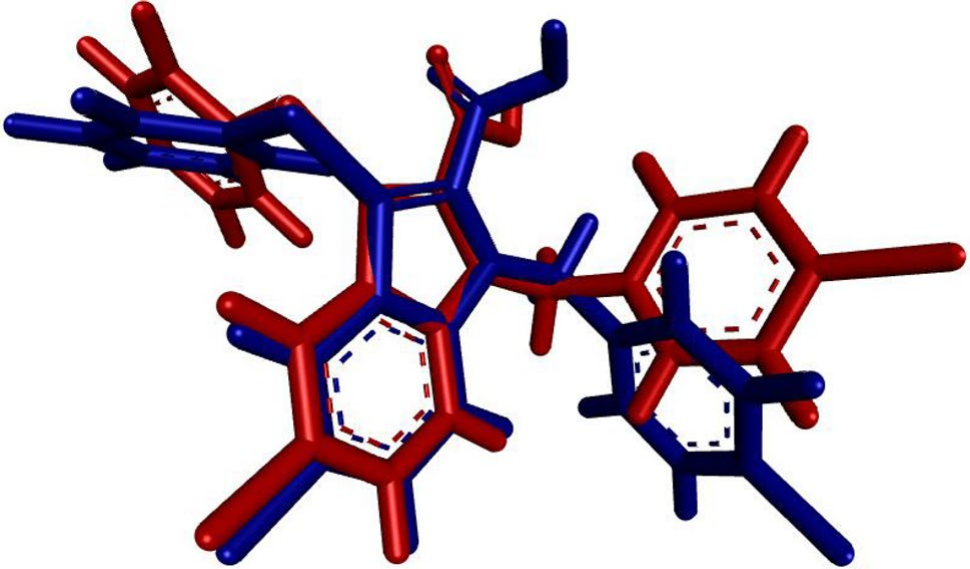
Superimposed native and re-docked native ligand conformations, RMSD (= 0.368) value indicated the reliability of the docking protocol.

**Figure 6:**
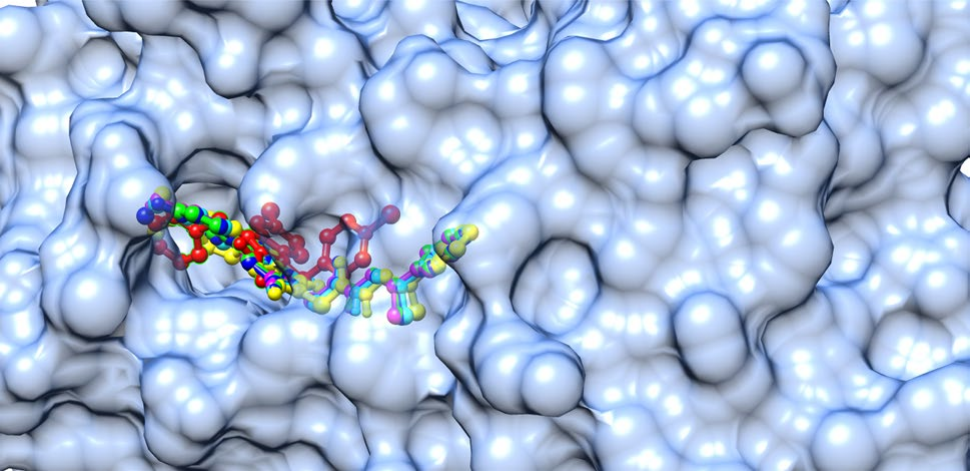
Native ligand (red); **5a** (green); **5b** (cyan); **5c** (blue); **5d** (magenta; and **5e** (yellow) engaged the same binding site of PPAR-γ.

The applied molecular docking protocol was successful in predicting the bioactive conformations of all investigated ligands. As shown in Fig. 5, the RMSD between the native and redocked native ligands was calculated to be 0.368 Å, indicating that the docking protocol was reliable. Figure 6 shows that all the titled compounds (**5a–e**) under investigation engaged the same binding site as the native ligand nTZDpa, a partial agonist. Compounds **5c** and **5d** were predicted to exhibit better binding affinities to PPAR-γ than the native ligand as shown in Table 4.

**Table 4:** Results of docking benzylidene-2,4-thiazolidinedione derivatives (**5a–e**) to PPAR-γ.

Compound ID	Binding energy (∆G: kcal/mol)	RMSD (Å)
nTZDpa (native ligand)	−9.8	0.368
**5a**	−9.8	0.767
**5b**	−9.7	0.788
**5c**	−10.1	0.721
**5d**	−10.0	0.730
**5e**	−8.3	0.794

**Figure 7:**
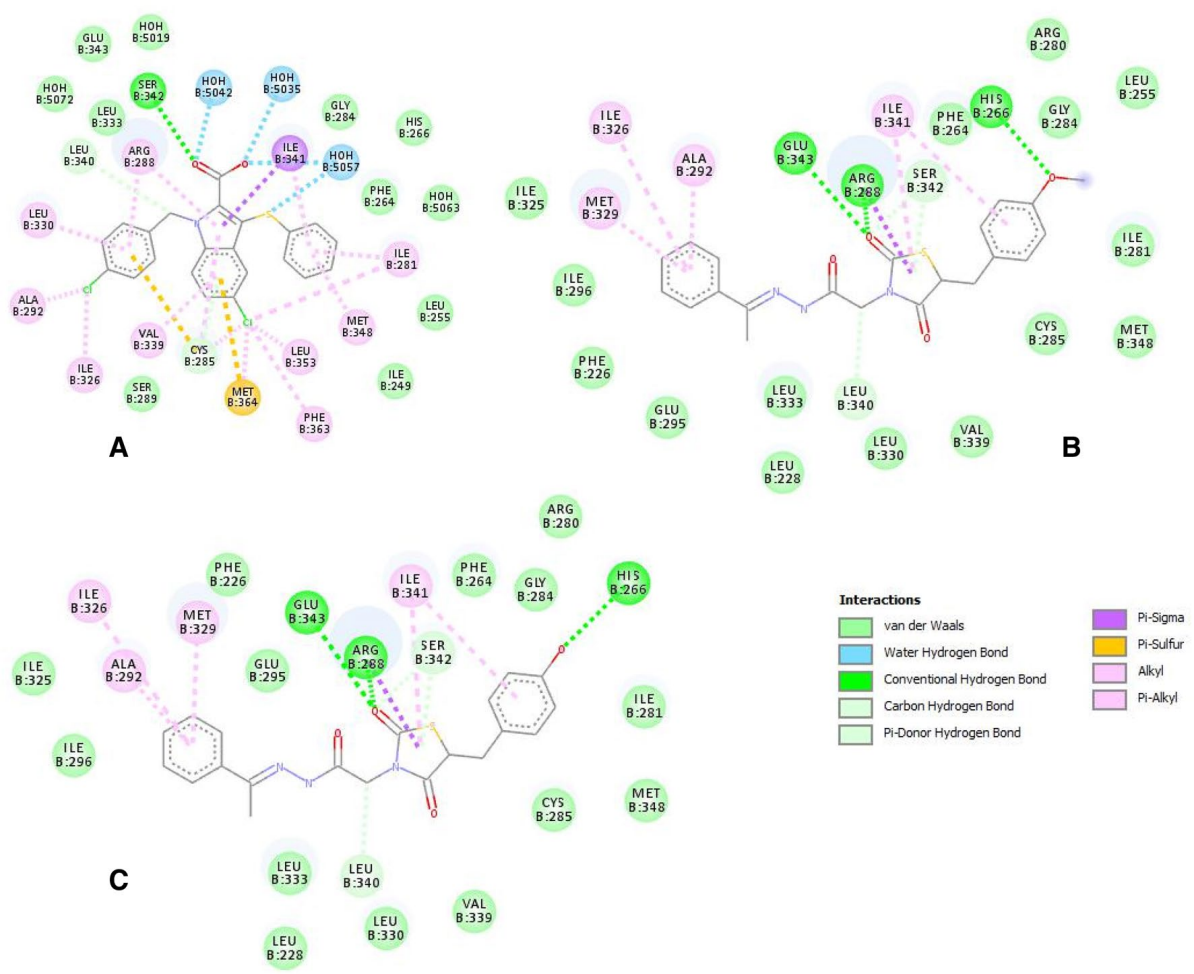
Two-dimensional interaction diagrams of (**A**) nTZDpa, partial agonist; (**B**) compound **5c**; (**C**) compound **5d** with PPAR-γ.

The binding interactions of the native and the top two ligands (**5c** and **5d**) were probed further to understand the nature of these binding interactions and the role of the ligand’s structural motifs in enhancing the binding to PPAR-γ. Figure 7A–C display the 2D interactions of the native ligand and compounds **5c** and **5d**, respectively.

PPAR-γ full agonists interact and stabilize the helix-12; however, partial agonists bind to alternative binding sites such as the helix 3 and β-sheet of the PPAR-γ ligand-binding domain^20^. The foremost interactions for a PPAR-γ partial agonist are hydrogen bonds with Ser342 of β-sheet, Arg288 of helix-5, hydrophobic interactions with Ile341 of β-sheet, and van der Waals interactions with the Cys285 of the helix 3 of PPAR-γ ligand-binding domain. The main difference observed between nTZDpa and compounds **5c** and **5d** was that the hydrophobic interactions contributed chiefly to nTZDpa binding, and the hydrogen bonds contributed significantly to the binding of compounds **5c** and **5d** to PPAR-γ. This finding suggested that the hydrogen bonds were involved in the binding of the test compounds to the protein, resulting in their lower binding energy than nTZDpa. All the characteristic features of partial agonist binding were predicted to be involved in the binding of compounds **5c** and **5d**. These compounds attained an extended conformation inside the binding site, facilitating the electrostatic hydrogen bonds with Arg288 rather than Ser342. The keto oxygen atom of the TZD ring played a crucial role in the binding to Arg288. Two π-donor hydrogen bonds were established with Ser342 by the heterocyclic thiazolidine ring and a keto group, elucidating the importance of the thiazolidinone ring in the receptor binding and its partial agonistic property. This interaction was in accordance with a recent report on PPAR-γ partial agonists^21^.

Hydrophobic interactions with Arg288, Ala292, Ile326, Met329, and Ile341 and van der Waals interactions with Cys285, Leu330, and Leu333 also significantly influenced the binding and partial agonist activity of the test compounds. The phenolic oxygen in compounds **5c** and **5d** participated in hydrogen bonding with His266, which was absent in the case of nTZDpa. The investigated compounds did not occupy the newly identified diphenyl pocket on the PPAR-γ ligand-binding domain^21^. Both compounds displayed similar interactions with PPAR-γ, except the van der Waals interaction of the methyl ether in compound **5c** with Leu255 residue. The hydrogen bond interactions of **5c** and **5d** with His266 were intriguing because the nTZDpa analog BVT.13 has been shown to interact in a similar manner with His266 and BVT.13. This partial agonistic activity is not dependent on H12 binding or stabilization but on the stabilization of β sheet^22^. Moreover, docking results indicated that compounds **5c** and **5d** did not engage the highly motile binding pocket surrounding β1, H2’, Ω-loop, and β2–β4 sheets made up of the residues Phe247, Ile249, Ile341, Gly344, Gly346, Met348, Thr349, and Arg350. Binding to the abovementioned binding site resulted in the hyperactivation of PPAR-γ^23^. Therefore, compounds **5c** and **5d** were identified as partial agonists of PPAR-γ.

The structure–activity relationship deduced from docking suggested that the conversion of the phenol (**5d**) to its methyl ether derivative (**5c**) slightly increased the binding affinity. However, similar electrostatic hydrogen bond interactions with the receptor were predicted because the oxygen atom of these groups was essential for the formation of hydrogen bond as the hydrogen bond acceptor, not the donor hydrogen. Chlorine (**5b**) and dimethylamino (**5e**) substituents on the phenyl ring did not improve the binding affinity. The thiazolidine ring and a keto group on the thiazolidine ring were important for binding to the receptor. The two terminal aromatic phenyl rings also played a crucial role in the PPAR-γ partial agonistic activity of **5c** and **5d**. The prototype of antihyperglycemic drugs is TZDs such as rosiglitazone and pioglitazone, which act as full agonists of the PPAR-γ receptor. However, they suffer from the disadvantages of causing heart failure, weight gain, edema, and obesity as side effects. PPAR-γ partial agonists are beneficial over full agonists because they exhibit the dose-related weak transactivation of the receptor by binding to different binding sites and utilizing different coactivators, leading to their distinguished effects^24^. Molecular docking results suggested that the partial PPAR-γ agonistic activity of compounds **5c** and **5d** were better than that of the native ligand. These findings were in good correlation with the in vivo results supporting the successful design of novel benzylidene 2,4-thiazolidinedione derivatives as PPAR-γ partial agonists for type 2 DM.

### Protein–ligand interaction-based pharmacophore model analysis

**Figure 8:**
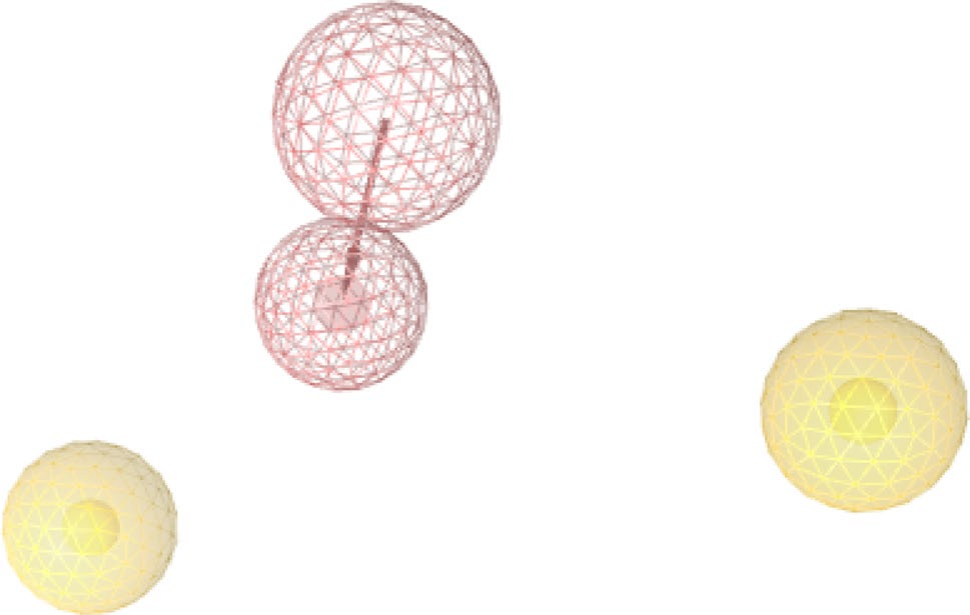
3D protein–ligand interaction-based pharmacophore model generated from the binding modes of newer benzylidene-2,4-thiazolidinediones. The pink colour depicts a hydrogen bond interaction through an acceptor group and the yellow colour depicts the hydrophobic interactions.

A 3D pharmacophore model based on protein–ligand interaction summarizes the structural features that are unique to the active conformation of the ligand bound to the binding site of the protein in a 3D space^19^. Figure 8 shows the 3D pharmacophore model generated from the characteristic structural features of compounds **5c** and **5d**. The generated 3D fingerprint may be considered as the lead structure that defines the pharmacophoric features necessary for partial PPAR-γ agonist activity. One hydrogen bond acceptor moiety and two hydrophobic moieties are essential for partial PPAR-γ agonism. The model also illustrated the distance and steric constraints between the protein and ligand. In the Ligand Scout software, the default distance constraint was 2.2–3.8 Å for hydrogen bond interactions and 1.0–5.9 Å for hydrophobic interactions. The rings around the structural features represent the exclusion coat that depicts the sterically forbidden area inside the binding pocket. Pharmacophore models are used as filters in high-throughput virtual screening^25^. Thus, this study created a 3D pharmacophore model with potential applications in the discovery of PPAR-γ partial agonists.

### Molecular dynamic simulation studies

**Figure 9:**
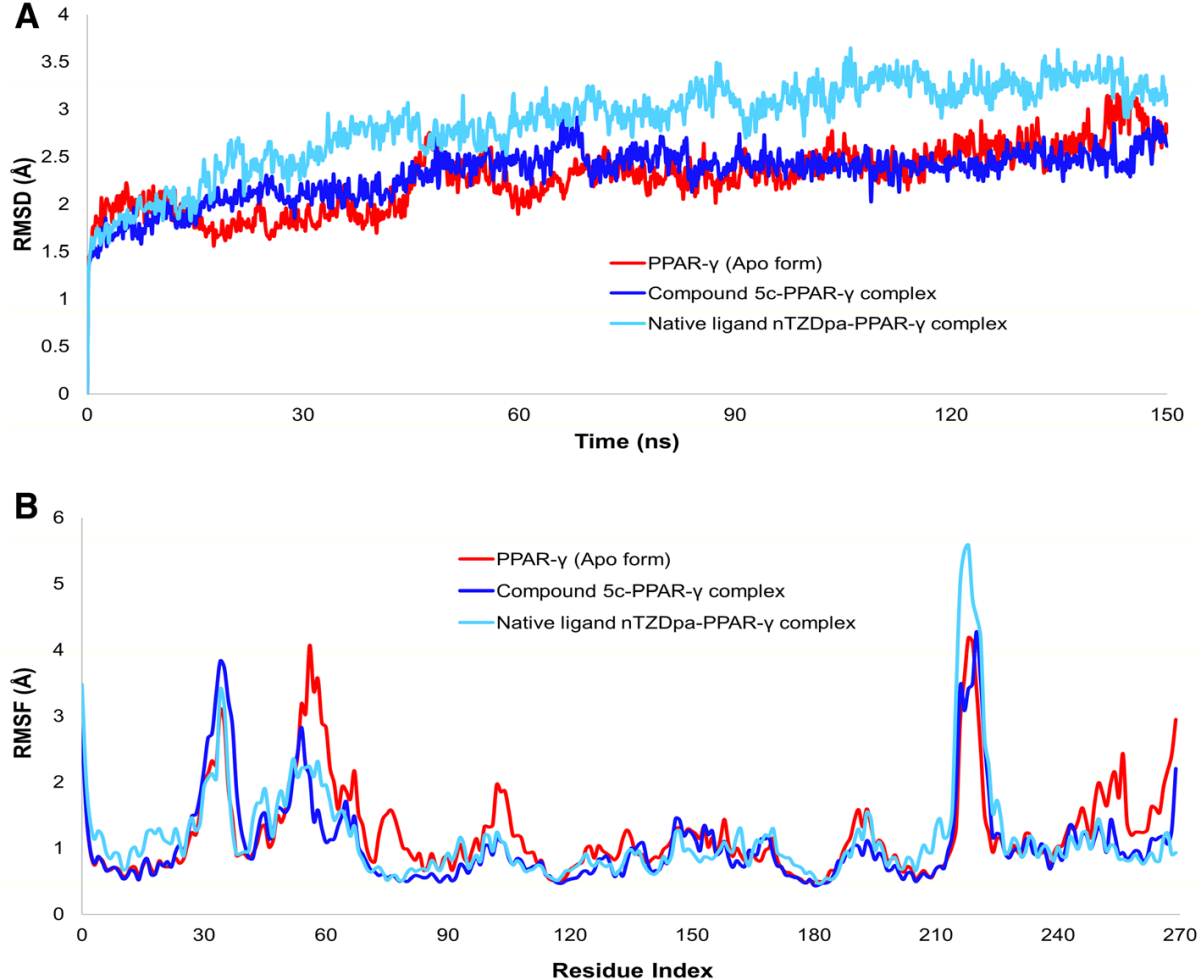
(**A**) RMSD of Cα atoms of PPAR-γ in apo form (red line) and in complex with compound **5c** (blue line) or nTZDpa (sky blue line); (**B**) RMSF of Cα atoms of PPAR-γ in apo form (red line) and in complex with compound 5c (blue line) or nTZDpa (sky blue line) during 150 ns MD simulation.

The stability of intermolecular interactions between PPAR-γ and compound **5c** was investigated using MD simulation**.** The conformation of the compound 5c-PPAR-γ complex with the lowest binding energy in the docking experiment was chosen. As a control, PPAR-γ protein in an unbound state and native partial agonist nTZDpa-PPAR-γ complex were also simulated for comparative analysis. The structural stability of the compound **5c** and PPAR-γ protein complex was evaluated by computing the root-mean-square deviations (RMSD) and root-mean-square fluctuations (RMSF) of the protein backbone atoms, as shown in Fig. 9**.** Compound 5c-PPAR-γ complex had an average RMSD value of 2.31 Å, whereas the nTZDpa-PPAR-γ complex and the unbound PPAR-γ protein exhibited an average RMSD value of 2.87 Å and 2.27 Å, respectively. The RMSD results demonstrated greater fluctuation of the nTZDpa-PPAR-γ complex than the compound 5c-PPAR-γ complex **(**Fig. 9A). The RMSD of compound **5c** indicated that it achieved a stable conformation starting from 70 ns and retained stability up to 150 ns of MD simulation.

The fluctuations of apo and compound **5c** or nTZDpa-bound protein structures were calculated on a residue basis by selecting carbon-alpha atoms. The Root mean square fluctuation (RMSF) illustrates the dynamics of each atom moving because of numerous hydrogen and hydrophobic connections with nearby residues. The unique conformational modes of interactions are a result of the position variation of the ligand molecules. As seen in Fig. 9B, the RMSF plot of the compound 5c-PPAR-γ complex exhibited less fluctuation and a more stable assembly than the apo or nTZDpa-PPAR-γ complex. The average RMSF values for the apo form and protein–ligand complexes of compound **5c** and nTZDpa were calculated to be 1.23 Å, 1.07 Å and 1.17 Å, respectively, for all amino acids of PPAR-γ protein over 150 ns simulated trajectories.

**Figure 10:**
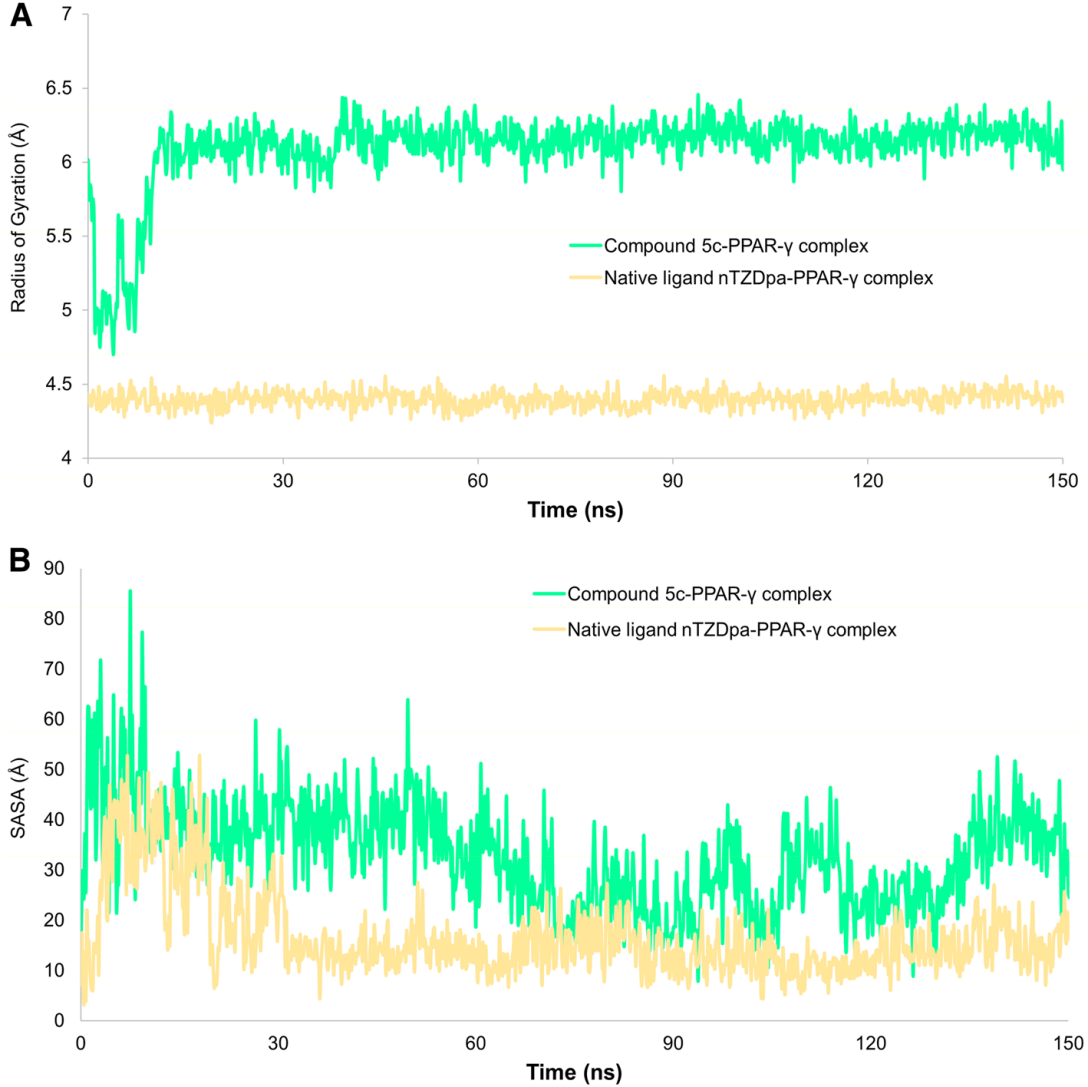
(**A**) Values of radius of gyration (rGyr); (**B**) solvent-accessible surface area (SASA) of the compounds **5c** and nTZDpa bound to PPAR-γ protein during 150 ns MD simulation.

The root mean square distance between the center of mass of each atom in the protein backbone is used to calculate the radius of gyration (rGyr). The rGyr of the compound 5c-PPAR-γ complex concerning the protein backbone is shown in Fig. 10A**.** The greater spreading area of the protein backbone's atoms accounts for the higher gyration values. The lower rGyr value, on the other hand, denotes the compactness of the protein backbone structure and the stability of the complex. The values of rGyr range between 4.70 and 6.46 Å in compound 5c-PPAR-γ complex whereas the rGyr ranged between 4.24 and 4.56 Å for nTZDpa-PPAR-γ complex, as shown in Fig. 10A, suggesting a stable and compact binding of compounds to PPAR-γ. The solvent-accessible surface area (SASA) of a protein is determined by the surface area accessible to the surrounding solvent molecules, which represents the state of folding compactness or unfolding, affecting the complex's stability. Figure 10B shows the SASA values for the compound 5c-PPAR-γ complex as a function of time.

**Figure 11:**
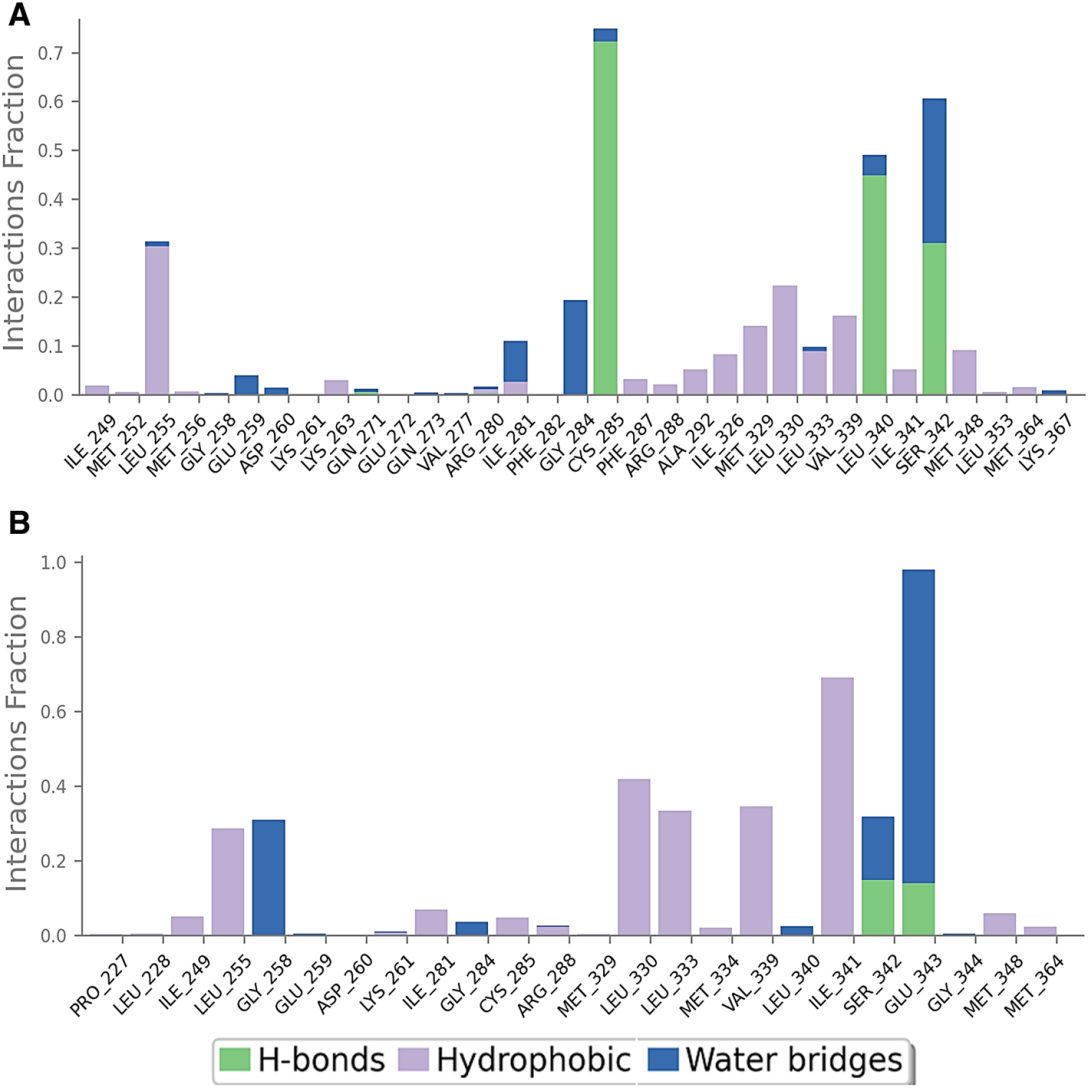
Intermolecular interactions of (**A**) compounds **5c**; (**B**) nTZDpa with PPAR-γ, monitored throughout the simulation period of 150 ns. H-bonds, hydrophobic, and water bridge interactions are grouped by type and presented in a bar diagram.

**Figure 12:**
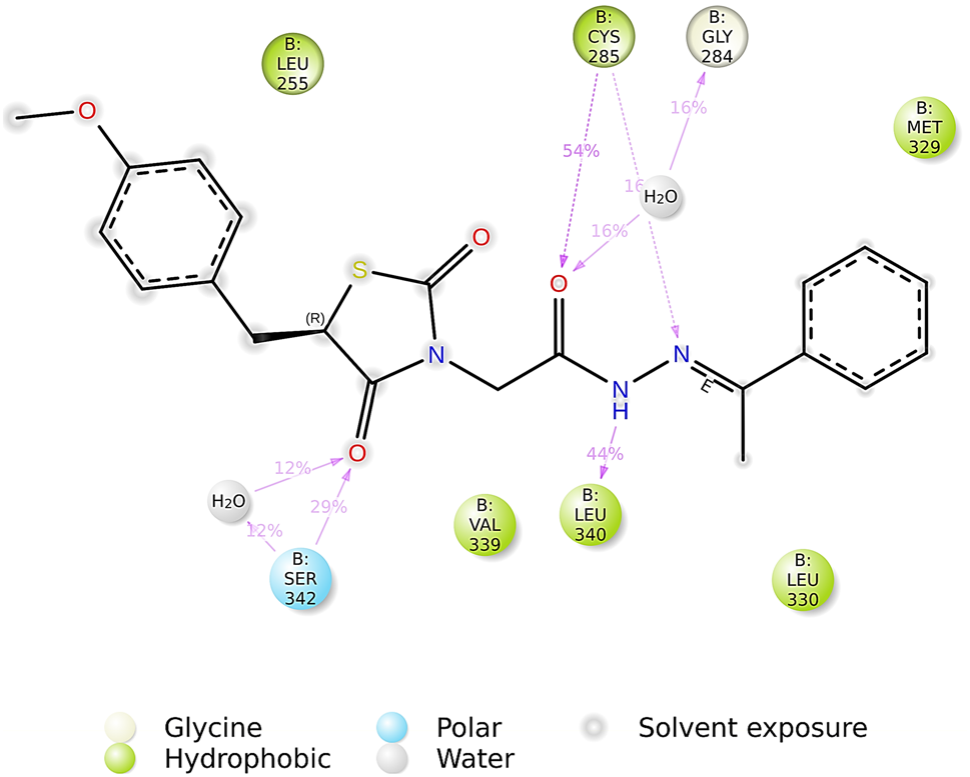
2D depiction of nature and stability of binding interactions of compound **5c** with PPAR-γ, observed throughout the MD simulation period of 150 ns.

**Figure 13:**
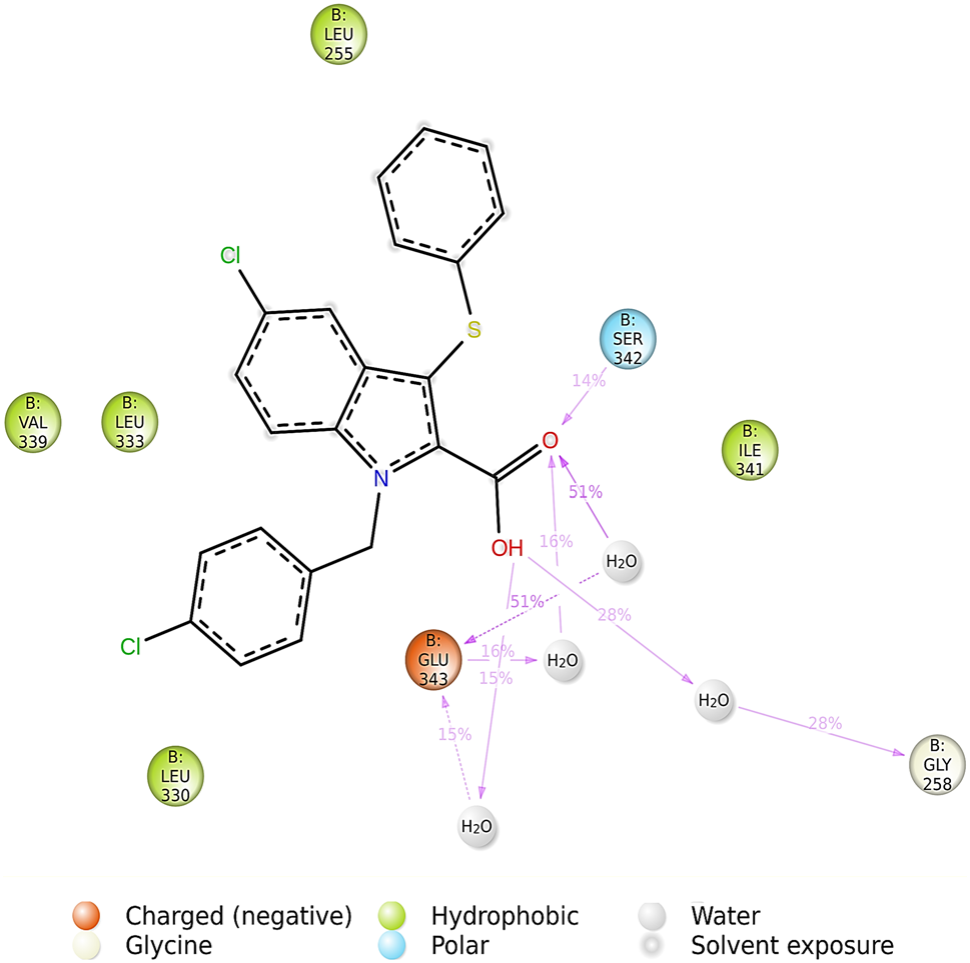
2D depiction of nature and stability of binding interactions of nTZDpa with PPAR-γ, observed throughout the MD simulation period of 150 ns.

Figure 11 summarizes the intermolecular interaction fractions of compound **5c** and the native ligand nTZDpa with PPAR-γ. More number of hydrogen bonds and hydrophobic interactions were predicted in compound 5c-PPAR-γ complex than in nTZDpa- PPAR-γ complex. Furthermore, the nature of binding interactions and their stability were analyzed from the extracted trajectories of MD. The 2D depiction of binding interactions in compound 5c-PPAR-γ complex and nTZDpa- PPAR-γ complex is shown in Figs. 12 and 13, respectively.

Figure 12 reveals the involvement of solvent bridges in facilitating polar hydrogen bond interactions between compound **5c** and PPAR-γ, which docking failed to predict. Moreover, MD unveiled a hydrogen bond interaction between keto oxygen of thiazolidinedione moiety and Ser342 alike nTZDpa. There are slight differences in the binding role of polar functional groups of compound **5c** and the binding residues of PPAR-γ predicted in docking and MD, but the number of hydrogen bonds predicted by docking and MD correlated well. The hydrogen bonds formed by compound **5c** with Cys285, Leu340, and Ser342 were stable and existed for 54%, 44%, and 29% of the time of MD. Figure 13 shows the stability of one hydrogen bond formed by nTZDpa with Ser342 (14% of the time) and two hydrogen bonds with Glu343 (51% of the time).

The supplementary Fig. S1, is the timeline graph delineating the interactions such as H-bonds, hydrophobic, ionic, and water bridges between compound **5c** and PPAR-γ residues during the simulation period of 150 ns. A deeper shade of orange indicates specific residues making more than one contact with the ligand. Fig. S2 is the timeline graph of nTZDpa presenting its various interactions with PPAR-γ. We have also supplied Video S3 which shows the movements of compound **5c** inside the binding site of PPAR-γ during MD simulation. In summary, MD simulations indicated that compound **5c** existed in a stable conformation inside the PPAR-γ binding site establishing stable hydrogen bonds with critical residues, confirming its potential to serve as partial agonist of PPAR-γ which requires further experimental validations.

## Conclusions

The developed benzylidene 2,4-thiazolidinediones proved to be promising antidiabetic compounds that partially agonize the PPAR-γ receptors. The results of in vivo and in silico investigations were in agreement with those from the structure–activity relationship studies, leading to the identification of derivatives with comparable antidiabetic activity to the standard drug rosiglitazone. The molecular docking and molecular dynamics studies suggested that the title compounds bind to the same location as the native ligand, acting as a partial agonist at the receptor. The title compounds mainly interacted with the receptor via hydrophobic interactions; the introduction of a hydrogen bond acceptor, such as oxygen, to the compound resulted in hydrogen bonding and increased binding energy. All the required characteristic features of a partial agonist were present in the most active tested compounds, suggesting their partial PPAR-γ agonistic mechanism. Further specific in vitro and ex vivo molecular level studies are required to establish the exact mechanism. The most active compounds of this series can act as potential leads whose structure can be modified to further improve their efficacy.

### Supplementary Information

Supplementary Figures.

Supplementary Video 1.

## Data Availability

The datasets used and/or analyzed during the current study are available from the corresponding author upon reasonable request.

## Abbreviations

Ala: Alanine
ANOVA: Analysis of variance
Arg: Arginine
BGL: Blood glucose levels
Cys: Cysteine
DM: Diabetes mellitus
DMSO: Dimethyl sulfoxide
FBG: Fasting blood glucose
FT-IR: Fourier transform-infrared spectrometry
Gly: Glycine
Glu: Glutamic acid
His: Histidine
ICH: International Conference on Harmonization
Ile: Isoleucine
LD_50_: Median lethal dose
Leu: Leucine
MD: Molecular dynamics
Met: Methionine
NMR: Nuclear magnetic resonance
nTZDpa: Non-thiazolidinedione PPAR-γ partial agonist
OECD: Organization for Economic Cooperation and Development
OGT: Oral glucose tolerance test
*p*: Level of significance
PDB: Protein data bank
Phe: Phenylalanine
PPAR-γ: Peroxisome proliferator-activated receptor-γ
rGyr: Radius of gyration
RCSB: Research Collaboratory for Structural Bioinformatics
RESPA: Reversible reference system propagator algorithm
RMSD: Root-mean-square deviation
RMSF: Root-mean-square fluctuation
SEM: Standard error of the mean
Ser: Serine
SASA: Solvent-accessible surface area
SPPARM: Selective PPAR-γ modulators
STZ: Streptozotocin
TG: Triglyceride
Thr: Threonine
TLC: Thin-layer chromatography
TZD: Thiazolidinediones
