# Supplementary material for: Synthesis, molecular docking, and in vivo antidiabetic evaluation of new benzylidene-2,4-thiazolidinediones as partial PPAR-γ agonists
Source: Sci Rep. 2023 Nov 14;13:19869. doi: 10.1038/s41598-023-47157-x (PMC10645977; doi:10.1038/s41598-023-47157-x)
Supplement: Supplementary file 1 — Supplementary Figures. [file 41598_2023_47157_MOESM1_ESM.docx]

**Submission ID:** 21bd5688-62b1-484b-8864-4b8ceea68817-R3

**Title: Synthesis, molecular docking, and in vivo antidiabetic evaluation of new benzylidine 2,4-thiazolidinediones as partial PPAR-γ agonists**


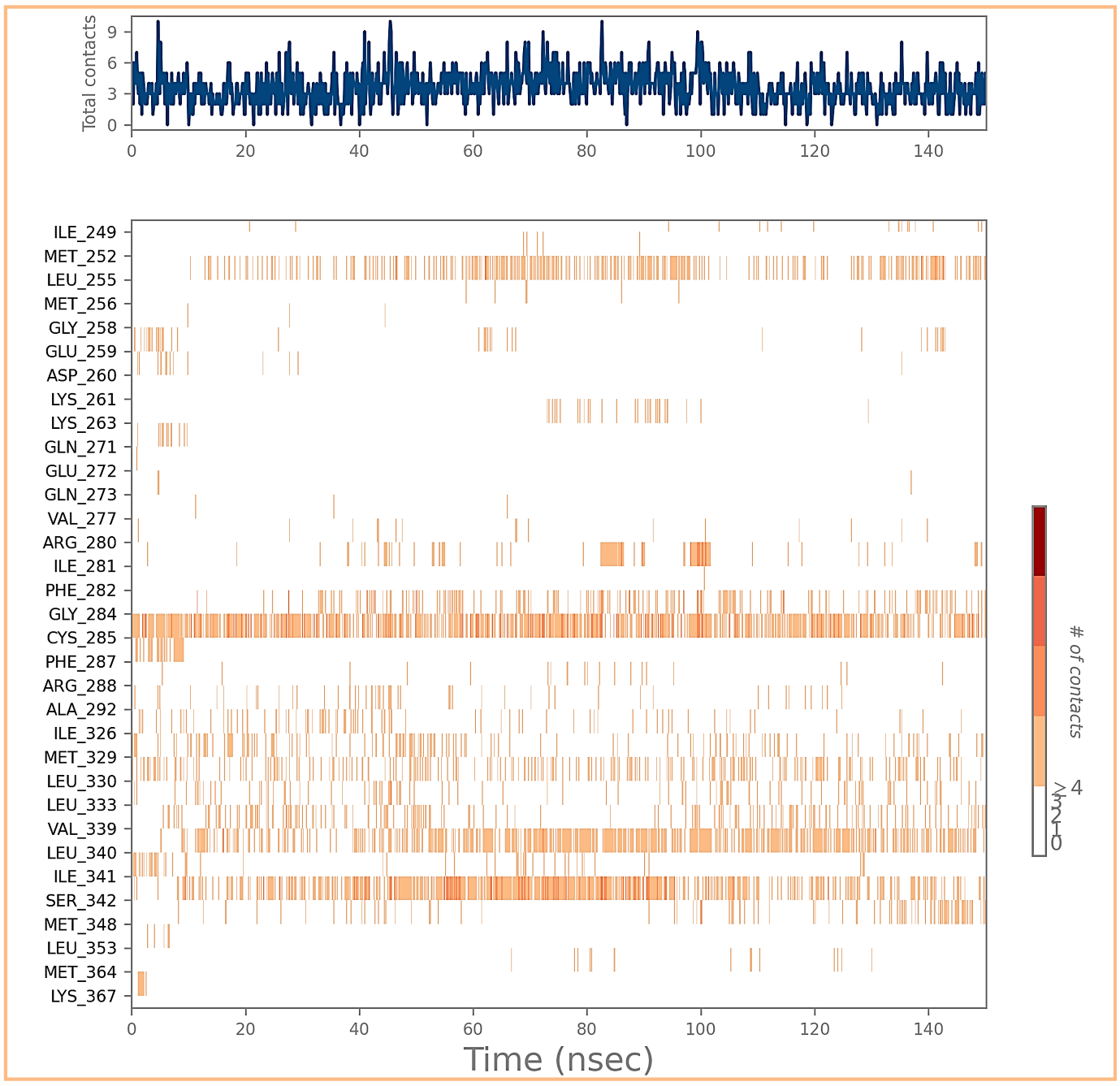


**Figure S1-** Timeline graph delineating the interactions such as H-bonds, hydrophobic, ionic, and water bridges between compound 5c and PPAR-γ residues during the simulation period of 150 ns. A deeper shade of orange indicates specific residues making more than one contact with the ligand.


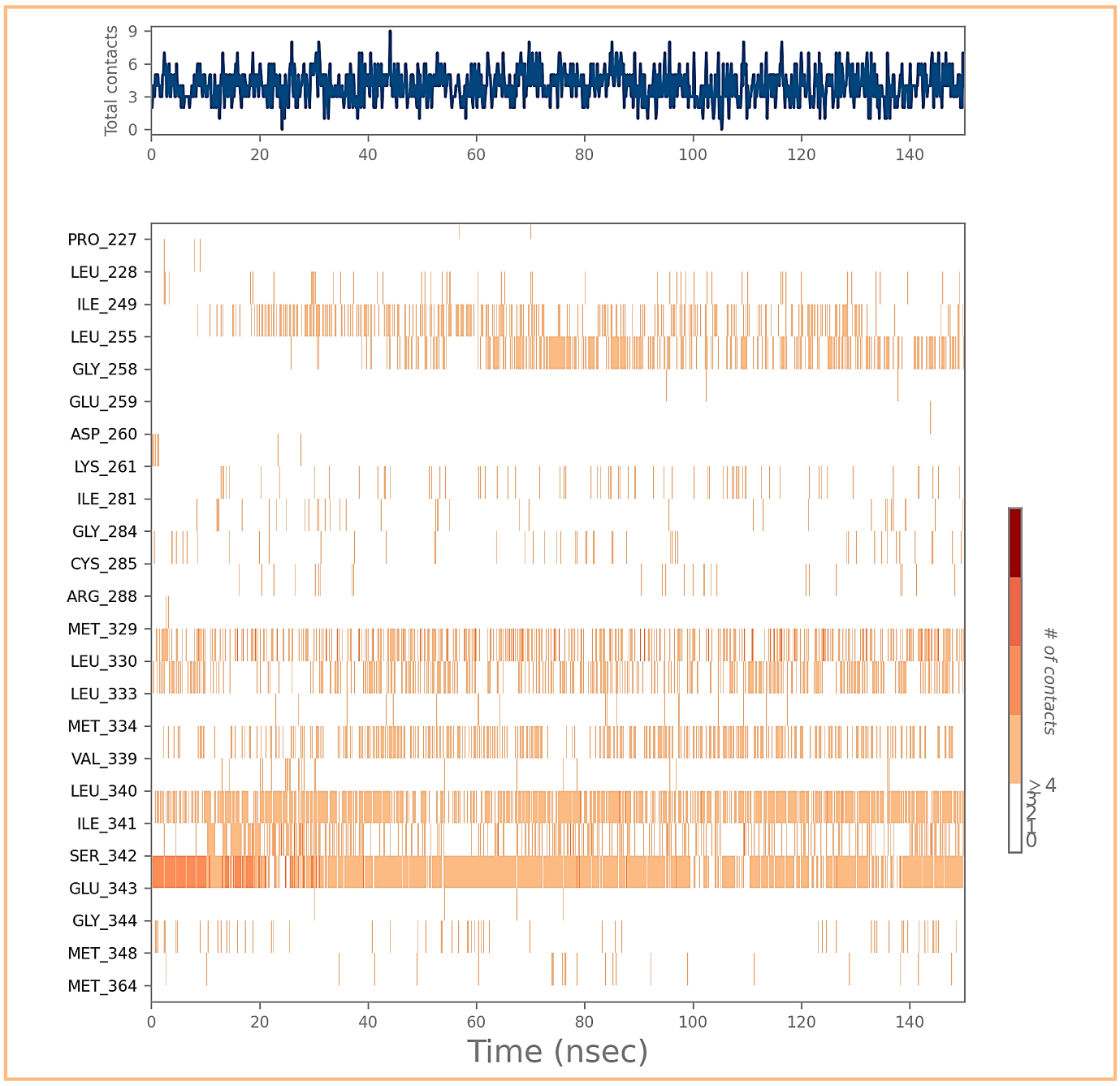


**Figure S2**- A timeline depicts the interactions such as H-bonds, hydrophobic, ionic, and water bridges between compound nTZDpa and PPAR-γ residues during the simulation period of 100 ns. A deeper shade of orange indicates specific residues making more than one particular contact with the ligand.
